# Prediction of Transcription Factor Binding Sites Using a Combined Deep Learning Approach

**DOI:** 10.3389/fonc.2022.893520

**Published:** 2022-06-03

**Authors:** Linan Cao, Pei Liu, Jialong Chen, Lei Deng

**Affiliations:** School of Computer Science and Engineering, Central South University, Changsha, China

**Keywords:** transcription factor binding sites, attention mechanism, positional embedding, deep learning, DNA

## Abstract

In the process of regulating gene expression and evolution, such as DNA replication and mRNA transcription, the binding of transcription factors (TFs) to TF binding sites (TFBS) plays a vital role. Precisely modeling the specificity of genes and searching for TFBS are helpful to explore the mechanism of cell expression. In recent years, computational and deep learning methods searching for TFBS have become an active field of research. However, existing methods generally cannot meet high performance and interpretability simultaneously. Here, we develop an accurate and interpretable attention-based hybrid approach, DeepARC, that combines a convolutional neural network (CNN) and recurrent neural network (RNN) to predict TFBS. DeepARC employs a positional embedding method to extract the hidden embedding from DNA sequences, including the positional information from OneHot encoding and the distributed embedding from DNA2Vec. DeepARC feeds the positional embedding of the DNA sequence into a CNN-BiLSTM-Attention-based framework to complete the task of finding the motif. Taking advantage of the attention mechanism, DeepARC can gain greater access to valuable information about the motif and bring interpretability to the work of searching for motifs through the attention weight graph. Moreover, DeepARC achieves promising performances with an average area under the receiver operating characteristic curve (AUC) score of 0.908 on five cell lines (A549, GM12878, Hep-G2, H1-hESC, and Hela) in the benchmark dataset. We also compare the positional embedding with OneHot and DNA2Vec and gain a competitive advantage.

## Introduction

The interaction between protein and DNA plays a pivotal role in *in vitro* life activities, such as mRNA transcription, DNA replication, and immune response (1). Transcription factors (TFs) are proteins that bind to regulatory DNA sequences and mediate gene expression. TF binding sites (TFBSs), also called motifs, typically range from a few to about 20 base pairs (bps) and are a type of DNA functional site. TF binds specifically to TFBS. Accurately finding the TFBS in the DNA sequence is essential for deciphering the mechanism of gene expression and understanding the life expression *in vitro* and drug design (2).

Studying the characteristics of TFBSs is a process of searching for subsequences with binding characteristics from the massive DNA sequence data. Unfortunately, traditional biological experiments are not only challenging to process massive amounts of data but also expensive and time-consuming. With the development of high-throughput technology, massive amounts of reliable experimental data can be obtained through *in vitro* experiments. These data contain potential TFBS sequences and provide convenience for obtaining TFBSs based on computational methods (3–5). MEME (4) searches for TFBS in DNA sequences by scoring the DNA sequences and then recursively selecting the sequences most likely to have motifs. AlignACE (5) computes possible sequences of TFBS based on Gibbs sampling. The common point of these algorithms is to use ChIP-seq high-throughput experimental data and statistical calculation methods to find potential TFBS, which has the characteristics of a large deviation of calculation accuracy. Because high-throughput experiments cannot accurately find the DNA subsequences where TFBSs are located under high-precision requirements, some sequence-based feature extraction methods have been proposed to solve the first step in motif searching. In past decades, position weight matrix (PWM) (1), OneHot (6), and K-mer (7) are all DNA representation methods that have achieved good results.

For the past few years, deep learning methods have been widely applied quite in many fields like computer vision, natural language processing, and speech recognition, and these fields have achieved good results, etc. (8–10). Predicting the interaction of biological sequences such as DNA/RNA sequences and protein sequences, as a new subject, has continuously been a very active research field, in which deep learning also plays a decisive role (11–19). Deep learning approaches can learn features from large amounts of data. DeepBind (6) is an earlier deep learning-based model in the field of gene sequencing. It miraculously adopts CNN to extract gene features predicted by protein binding sites, thus reshaping the entire era of using convolution kernels to capture features. In (12, 14), by fine-tuning the network architecture of CNN, the validity of various networks to verify TFBS has been evaluated in terms of overall. DanQ (13), the one who tactfully used long short-term memory (LSTM) to improve the before-and-after dependency in gene features, further enhanced the performance in the task of quantifying gene sequence functions.

Although the methods based on deep learning have achieved significant results in discovering TFBS, at this stage, a more in-depth and comprehensive application still needs great improvement: 1) accurately embedding the DNA sequence has been decisive to promote the model’s performance. In previous studies, the traditional method such as OneHot (6) for encoding has been proposed as a promising, relatively achieved good performance, but it is difficult to improve due to its explosion of the consumption of computing resources when the OneHot embedding size increases. In (20), the NLP method has many applications in the field of DNA sequence and realizes distributed embedding representation. However, it was found that the position information contained in the DNA sequence was lost during use. 2) It has proven effective as an emerging method at predicting capabilities in successfully applying the attention mechanism for NLP. However, there is still an uneasy process with multiple challenges that need to be addressed before acquiring practical application potential, such as limited knowledge of an outstanding method to integrate it into the field of genes. In this work, we develop a combined deep learning approach that uses OneHot and DNA2Vec embedding to extract the hidden embedding from DNA sequences and apply CNN and bidirectional LSTM network (BiLSTM) with an attention mechanism to build the prediction model. Experimental results show that our proposed method predicts better than existing state-of-the-art methods and has good interpretability.

## Materials and Methods

### Datasets

High-throughput experiments produce a mass of protein-DNA binding datasets. We use ENCODE (Encyclopedia of DNA Elements), which offers TF cell type binding data analyzed by the ChIPseq method (21) to train and test our model. Zeng’s works (12) have completed the preprocessing part of the work. In the preprocessing work, the positive samples consisting of 101 bps were generated in the central region of each ChIP-seq peak. The negative sample is obtained by recombining the positive sequence with the matching length. We distinguish positive samples from negative samples based on whether TFBS can be found in the sequence. So the positive samples represent TFBSs, while negative samples do not have binding sites with a TF in the sequence. In this study, we adopted 50 datasets that were selected at random from 690 ChIP-seq datasets, including five cell lines (GM12878, H1-hESC, Hep-G2, Hela, and A549) as training sets and testing sets to measure the model performance. Of these data, 60% are used as the training set, 30% as the test set, and 10% as the verification set. In this article, our method runs as follows: first, we embed each DNA sample to get the position information and DNA sequence content features at the same time. Then we feed the DNA embedding to the attention-based model to get the final prediction.

### Problem Statements

**Figure 1:**
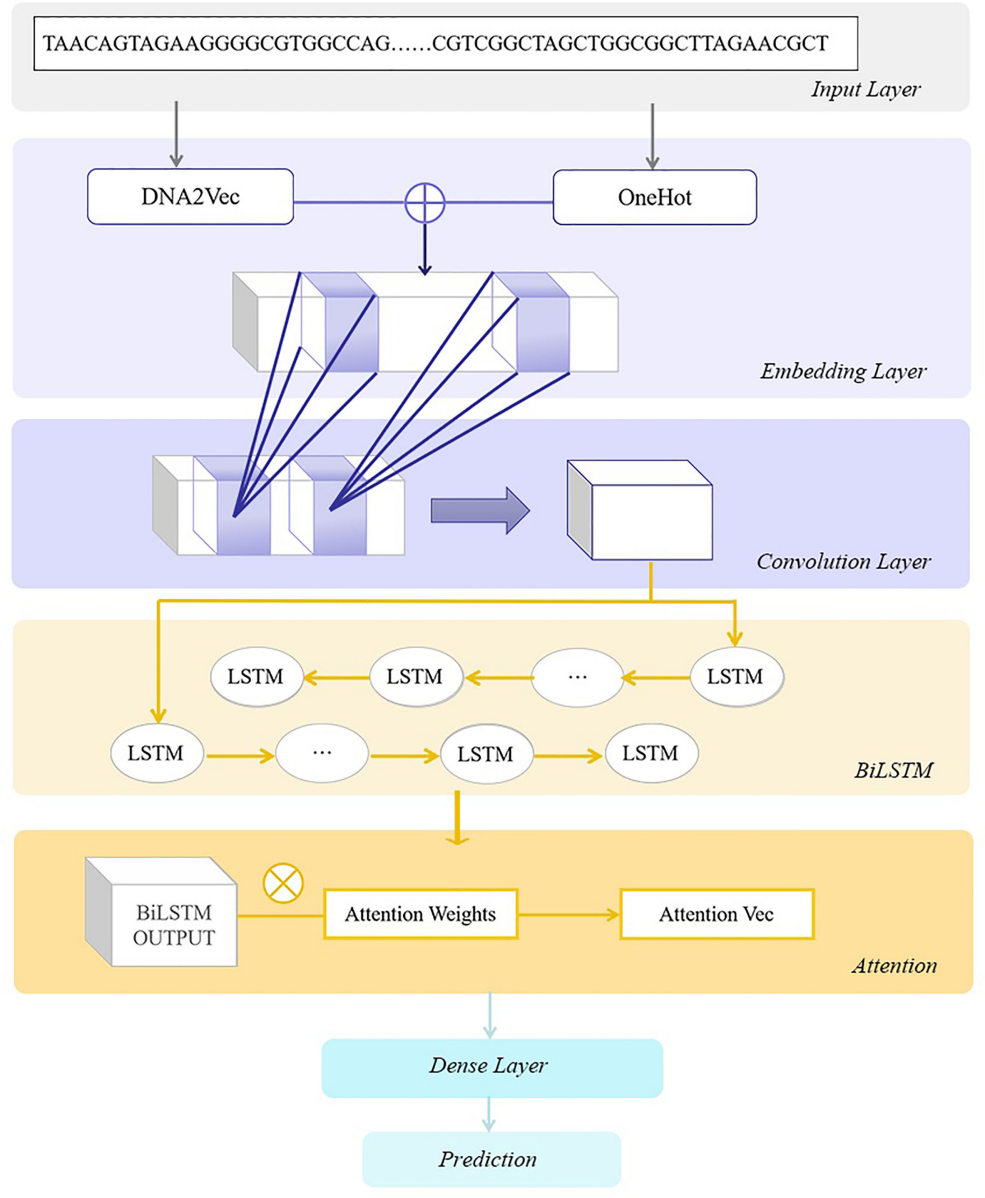
The architecture of DeepARC. In the embedding layer, OneHot and k-mer encoding are used to generate position-based feature embedding from DNA sequences. Then convolution kernels are utilized to extract non-linear features. In the BiLSTM layer, we use a bidirectional long short-term memory network (BiLSTM) to capture the contextual dependencies of DNA sequences. Next, we use the attention mechanism to enhance the model**’**s prediction performance, and finally, the prediction results are obtained through the dense layer.

The problem of TFBS prediction can be expressed as follows. First of all, we divided all gene sequences into two categories based on whether TFBS could be found in the DNA sequence. The two categories are represented by label 0 or 1, which means that there is no TFBS or TFBS in the gene sequences, respectively. The embedded DNA sequences are expressed by ${\left\{X^{\left(i\right)},y^{\left(i\right)}\right\}}_{i=1}^{n}$ and input into the model where *X* ^(^ *^i^* ^)^ is the input DNA sequence data and *y* ^(^ *^i^* ^)^ shows the type of gene sequence. After that, we train the model DeepARC (**Figure 1**) on the training sets. Our goal is to obtain high-accuracy classification results in the testing sets and extract the consistent sequence features from a tremendous amount of gene information.

### Positional Embedding

**Figure 2:**
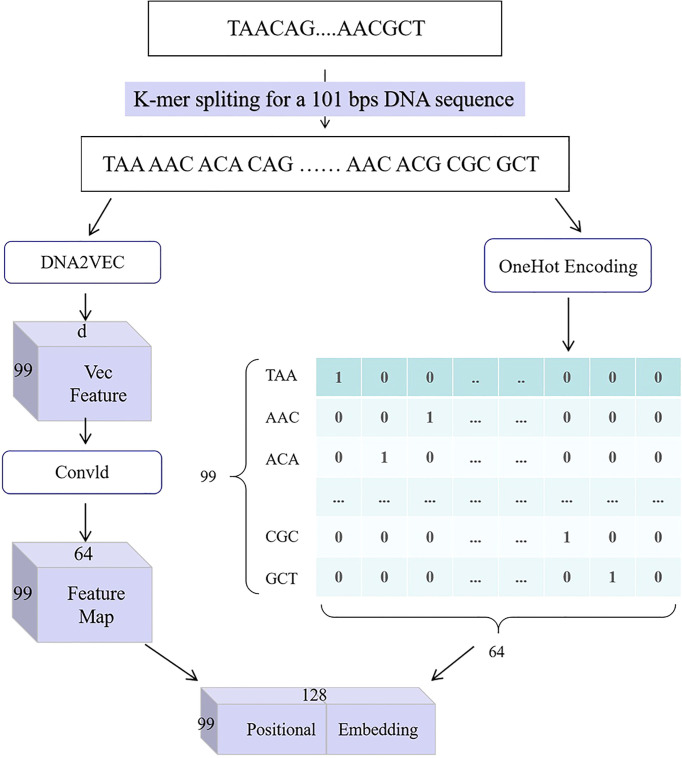
DNA positional embedding for an original DNA sequence with 101-bps length and 3-mer split.

1) OneHot encoding, also called one-bit efficient encoding, uses the N-bit status register to encode N states, each of which has its own independent register bit, and at any given time, only one bit in the code is valid and can be used to map characters to a unique encoding. As a simple and effective coding method, OneHot encoding has been widely used for indicating the state of a state machine, and there are many applications in bioinformatics and natural language processing (6, 14). In DeepBind, each fragment of gene sequence is regarded as a feature and encoded by OneHot in a special way. However, there are some drawbacks to the OneHot encoding. Due to the simple and sparse characteristics of OneHot encoding and the assumption that different features are independent, the mutual relationship between different coding units will be lost, and the distance relationship between coding units will not be reflected. For data with some kind of continuous relationship, encoding with the OneHot method may result in a situation where the accuracy rate will be significantly reduced. In addition, the OneHot encoding dimension of each word is the size of the entire vocabulary. With the growth of embedded data, the dimension will become huge, and the coding will turn sparse, which will make the calculation cost very terrible.

2) In the field of molecular biology, mer represents a monomeric unit, and k-mer means a set of nucleotide strings with a length of k. Extracting k-mer from L-length DNA sequence can generate *L* − *k* + 1 fragments, and the association between different sequences after k-mer division can be preserved in these fragments. In WSCNNLSTM (17), instead of using OneHot coding, k-mer features of sequences are extracted. The association between sequences is still maintained in k-mer after the gene sequence is divided into k-mer. Therefore, WSCNNLSTM has better performance in TFBS classification work. A new method is proposed in DNA2Vec that can calculate the distribution representations of k-mer with variable length (20), which apply to NLP to biological sequence information.

3) In this paper, we present a gene position embedding method, which transforms gene sequence into a characteristic matrix, as shown in **Figure 2**. It uses a combination of the OneHot encoding method, the DNA2Vec method, and the convolutional module. The workflow of this method is as follows: first, the input gene sequence, *S* = (*s*_1_, *s*_2_, …, *s_l_*), is divided into *L*–*k*+1 sequence features by k-mer cutting method, which is called *Z_mer_* = (*z*_1_,*z*_2_,…,*z_L_*_–_ *_k_*_+1_). Next, we use OneHot encoding on the mer to get the unique location information called, as well as adopt DNA2Vec to get the context feature called *Z*_*vec*_∈*ℝ* ^(*L*−*k*+1)×*d*^, where *d* is the dimension of word embedding in the DNA2Vec. Because the feature dimension obtained by DNA2Vec is too high, we try to decrease the word embedding dimension to 4*^k^* by extracting features through a convolution module. Finally, we linked the high-order dependent features Z_OH_ and Z_vec_ to obtain DNA position embeddings $Z_{pe}\in {\mathbb{R}}^{\left(L-k+1\right)\times \left(2\times 4^{k}\right)}$ .

### Convolutional Neural Network

The convolutional neural network (CNN) is a sort of feedforward neural network with convolution calculation and depth architecture (18). It is successfully used in image recognition, video analysis, natural language processing, drug discovery, and other fields and has achieved good results (22, 23). The working process of CNN is usually to input image information; then pass a battery of convolutional layers, non-linear layers, pooling layers, and complete connection layers; and then get the final output result. Among them, the convolutional layer mainly has the function of feature extraction through the scan of the convolutional kernel, while the pooling layer primarily plays the role of feature selection and information filtering. Therefore, CNN greatly reduces network parameters and has translational invariant properties. In the field of bioinformatics, CNN was initially applied to deal with DNA sequence information in the DeepBind. After embedding the DNA sequence in some manner, it is disposed of in the shape of a graph in the network. CNN is able to extract multiple features through scanning different convolution kernels so that it can handle various downstream works.

Because of its strong feature extraction ability, CNN was used to capture TFBS in this study. The embedded gene sequence *Z_pe_* was input into the CNN model to get the extracted feature *C*. In our experiment, there are two submodules in the CNN model, and each submodule is composed of a convolution layer and a non-linear activation layer. Among them, the convolutional layer mainly plays the function of detecting TFBS to obtain features similar to TF-motif. The parameters of the model are set as follows: the size of the convolution kernel is 5, the padding is 2, and the step size is 1. The purpose of such a setting is to make the dimension of feature *C* to represent the constant length of the sequence and to be able to learn the features of the whole DNA sequence through BiLSTM. In order to prevent over-fitting of the model, the ReLU function is used in the non-linear activation layer. Finally, we extracted feature *C* through the CNN module.

### Bidirectional Long Short-Term Memory Networks

BiLSTM is a particular type of recurrent neural network (RNN), which has parameter sharing, Turing-complete, and memorability, so it has some advantages in learning non-linear characteristics of sequences. Compared with RNN, BiLSTM can handle the long-term dependency problem existing in RNN and can realize the real context-based consideration, so it also has higher accuracy (24). In terms of structure, based on the traditional RNN model, LSTM also adds a gate structure of forgetting gate, input gate, and output gate to control the information passing through the model, through the gate structure to control the input and output information flow, so as to solve the problem of long-term dependence in RNN. BiLSTM combines forward LSTM and backward LSTM for accurate context analysis. The following is the operation formula of the LSTM memory unit:


(1) $$f_{t}=\sigma \left(W_{xf}x_{t}+W_{hf}h_{t-1}+b_{f}\right),$$

(2) $$i_{t}=\sigma \left(W_{xi}x_{t}+W_{hi}h_{t-1}+b_{i}\right),$$

(3) $$c_{t}=f_{t}⊙c_{t-1}+i_{t}⊙tanh\;\left(W_{xc}x_{t}+W_{hc}h_{t-1}+b_{c}\right),$$

(4) $$o_{t}=\sigma \left(W_{xo}x_{t}+W_{ho}h_{t-1}+b_{o}\right),$$

(5) $$h_{t}=o_{t}⊙tanh(c_{t})$$

where *i*, *f*, *o*, *c*, and *h* represent the input gate, forget gate cell vector and hidden vector, respectively. *W* is the gate matrix, and *b* is the bias. The index *t* refers to the time step *σ* is the logistic sigmond function *tanh* is the active function to force the values to be between -1 and 1, and ⊙ denotes element-wise multiplication.

The DNA sequence is a series of letters used to represent the actual or hypothetical primary structure of DNA molecules carrying genetic information, which can be considered the mystery of life’s sequence language to some extent. The LSTM model has been introduced by DeeperBind (14) and DeepTF (25) to analyze the long-term dependence of DNA sequences. However, in this paper, we adopt BiLSTM to capture associations between successive gene sequences. BiLSTM is composed of forward propagating LSTM and backward propagating LSTM and can analyze forward and backward sequence information. Therefore, it has higher accuracy than LSTM. The input of the BiLSTM model is the feature generated by passing the convolution layer, and the output includes the output feature *P* and the hidden state information *h_n_*. It is worth noting that the sum output by the forward model and the backward model of BiLSTM is the final output feature at the *i*th position, and the formula is as follows:


(6) $$h_{i}=\left[\vec{h_{i}}⊕\overset{\leftarrow }{h_{i}}\right]$$

### Attention Mechanism

In essence, the attention mechanism in deep learning is analogous to the selective visual attention mechanism in mankind, and the major objective is to select more important information for the current work goal from massive details. The attention mechanism is a kind of resource allocation scheme, which is the principal method to deal with the trouble of information overload, and it is very suitable in the case of limited computing power. It allocates computing resources to more critical work and improves the utilization of resources. The self-attention mechanism has been used by BERT (26) to train natural language and has also obtained excellent results in text classification, machine translation, and other works. Attention mechanism has been used in the field of deep learning far and wide and achieved good results in named entity recognition, machine translation, and other fields (8–10, 19). Therefore, a soft attention mechanism was adopted in our experiment to focus attention on the TFBS we were looking for. The feature *P* and the hidden state h_n_ after the BiLSTM module are used as the input of the soft attention mechanism. The mer-level feature is merged into a sentence-level feature vector to generate the attention-weight vector (27). Finally, the DNA attention vector for the classified prediction can be calculated. The formula is as follows:


(7) M=tanh(H),

(8) $$\alpha =softmax(\omega^{T}M),$$

(9) $$\gamma =H\alpha^{T},$$

(10) $$h^{*}=tanh(\gamma )$$

where H∈*ℝ* ^*d* ^*w*^×*T*^, *d^w^* is the dimention of the word vectors, *ω* is a trained parameter vector, and *ω^T^* is a transpose. The dimention of *ω*, *α*, and *γ* is *d^w^*, *T* and *d^w^*, respectively.

### Dense Module

The dense module constituted by two layers of a fully connected neural network, one dropout layer core and one sigmoid function, is the last module of the whole model. The full connection layer mainly acts as a classifier to classify the input into several categories. However, since the full connection layer has too many parameters, we added a dropout layer to the back of the full connection layer to prevent the over-fitting of the model from improving the generalization ability of the model (28). We take the binary cross-entropy loss calculated by the prediction and the goal as the cost function of the model, and the formula is as follows:


(11) $$loss=-\frac{1}{N}\sum_{n=1}^{N}\left[y_{n}\log\;x_{n}+(1-y_{n})\log(1-x_{n})\;\right]$$

where *x_n_* is the prediction and *y_n_* is the goal.

## Results

DeepARC is an attention mechanism-based model for predicting the presence or absence of TFBSs on gene sequences. In the experiment, we randomly selected 50 datasets from ENCODE to conduct model training. To demonstrate the advantages of the model architecture and location embedding used in this article, we also compare it to similar approaches that are currently popular. So as to test the property of DeepARC, we will use the three most advanced algorithms in this field to carry out comparative experiments on the same dataset. In the following content, we will analyze the experimental results in detail. First, we introduce the evaluation indicators used in this experiment. Second, the advantages of our model and the advantages of our location-embedding approach are presented. Third, we mainly introduce the performance comparison of our method with existing excellent predictors. Finally, we explain the attention mechanism used in the article.

### Evaluation Measurements 

Due to the characteristics of this experiment, we decided to select five evaluation measurements—sensitivity (*Sen*), specificity (*Spe*), accuracy (*Acc*), Mathew’s correlation coefficient (*MCC*), and the area under the receiver operating characteristic curve (*AUC*)—to evaluate the prediction ability of our model (29). Their formula is as follows:


(12) Sen=TP/(TP+FN)

(13) Spe=TN/(TN+FP)

(14) Acc=(TP+TN)/(TP+FP+TN+FN)

(15) $$MCC=\frac{\left(TP\cdot TN-FP\cdot FN\right)}{\sqrt{\left(TP+FP)(TN+FN)(TP+FN)(TN+FP\right)}}$$

where *TP*, *TN*, *FP*, and *FN* are the number of true positives, true negatives, false positives, and false negatives, respectively.

### Performance Comparison With Other Model Frameworks

In this experiment, we used the data encoded by OneHot as input, adopted the Adam optimizer (30), and set the learning rate to 0.001 and the step size to 20 to show the performance of the model architecture. In addition, in order to reflect the excellence of the model mechanism, we compare the performance differences of CNN-BiLSTM, BiLSTM-Att, and DeepARC in the same input set. CNN-BiLSTM represents a model whose model architecture is CNN+BiLSTM, BiLSTM-ATT represents a model whose architecture is BiLSTM+attention mechanism, and CNN-BiLSTM-Att is the model architecture used by DeepARC. The results of the three cross-validation tests are considered to be the final model performance. **Table 1** shows the final performance comparison results for each model, and **Table 2** describes the other detailed parameter settings.

**Table 1:** Performance comparison of CNN-BiLSTM, BiLSTM-Attention, and CNN-BiLSTM-Att with OneHot embedding.

Dataset	Model	*Sen* (%)	*Spe* (%)	*Acc* (%)	*MCC*	*AUC*
	CNN-BiLSTM-Att	**80.66**	83.66	**82.16**	**0.644**	**0.901**
A549	CNN-BiLSTM	79.42	82.61	81.01	0.625	0.896
	BiLSTM-Att	74.13	**86.96**	80.55	0.616	0.887
	CNN-BiLSTM-Att	**81.05**	83.02	**82.04**	**0.641**	**0.902**
GM12878	CNN-BiLSTM	75.63	**86.51**	81.07	0.625	0.891
	BiLSTM-Att	73.90	85.58	79.74	0.600	0.880
	CNN-BiLSTM-Att	79.95	74.32	**77.13**	**0.545**	**0.860**
Hela	CNN-BiLSTM	**80.17**	73.81	76.99	0.543	0.858
	BiLSTM-Att	76.32	**75.94**	76.13	0.524	0.845
	CNN-BiLSTM-Att	81.47	84.50	**82.98**	**0.660**	**0.908**
Hep-G2	CNN-BiLSTM	**85.87**	77.99	81.93	0.641	0.906
	BiLSTM-Att	76.78	**86.18**	81.48	0.634	0.897
	CNN-BiLSTM-Att	81.26	**82.13**	**82.72**	**0.636**	**0.891**
H1-hESC	CNN-BiLSTM	**82.19**	81.31	81.25	0.629	0.883
	BiLSTM-Att	76.11	81.52	82.32	0.612	0.876

CNN, convolutional neural network; BiLSTM, bidirectional long short-term memory network; Sen, sensitivity; Spe, specificity; Acc, accuracy; MCC, Mathew’s correlation coefficient; AUC, area under the receiver operating characteristic curve.

The bold section indicates the best performing indicators in each dataset.

**Table 2:** Parameters setting of different models.

	CNN	CNN	-
Parameter	BiLSTM	BiLSTM	BiLSTM
	Att	–	Att
Learning rate	0.001	0.001	0.001
Epochs	20	20	20
Batch size	64	64	64
CNN layers	2	2	–
Kernel size	5	5	–
BiLSTM hidden size	16	16	32
Attention vec size	16	–	32
Dense neurons	16	32	32
Dropout	0.2	0.2	0.2
Optimizer	Adam	Adam	Adam

CNN, convolutional neural network; BiLSTM, bidirectional long short-term memory network.

As shown in **Table 1**, in the five datasets, our model (CNN-BiLSTM-Att) generally has the best performance and always has the highest score in Acc value, MCC value, and AUC value. First of all, by comparing the performance of CNN-BiLSTM-Att and BiLSTM-Att on the five datasets, it can be found that except for the Spe, CNN-BiLSTM-Att has better performance on other evaluation values, namely, Sen, Acc, and MCC were 5.43%, 1.36%, and 1.4% higher, as compared with BiLSTM-Att. It can be seen that CNN is used to find TFBS features and has a good effect. Next, we compared CNN-BiLSTM-Att with CNN-BiLSTM and found that CNN-BiLSTM-Att has Sen, Acc, and MCC of 0.22%, 0.95%, and 1.26% higher than CNN-BiLSTM on the 5 datasets. It can also be seen that the attention mechanism enhances the weight of the model on motif to effectively promote the performance of the model. Compared with other popular model frameworks, our model architecture obviously has higher performance and certain advantages. However, it can also be seen from the table that our model has a poor performance in SPE. The lower SPE may be due to the tendency of the model’s predicted samples to be positive.

### Performance Comparison Among Positional Embedding and Other Methods

In this part, we primarily analyze the property of positional embedding in the DNA embedding part. To more intuitively observe the advantages and disadvantages of the performance, we compare the positional embedding method with DNA2Vec and OneHot. On the basis of the research (7), we set 3-mer to implicitly capture the binding information, 3-mer splitting, and one stride in the embedding. Consistency is maintained by using the CNN-BiLSTM-Att in the previous section as the model for the experiment. Moreover, the hyperparameters and prediction methods of the three methods are consistent with the above methods. **Table 3** shows the experimental performance results.

**Table 3:** Performance comparison of OneHot, DNA2Vec, and positional embedding.

Dataset	Model	*Sen* (%)	*Spe* (%)	*Acc* (%)	*MCC*	*AUC*
	Positional embedding	**83.17**	83.74	**83.46**	**0.671**	**0.909**
A549	DNA2Vec	77.58	**85.64**	81.61	0.635	0.896
	OneHot	80.66	83.66	82.16	0.644	0.901
	Positional embedding	**81.31**	**83.48**	**82.40**	**0.650**	**0.905**
GM12878	DNA2Vec	80.81	81.25	81.03	0.623	0.895
	OneHot	81.05	83.02	82.04	0.641	0.902
	Positional embedding	**80.36**	**84.64**	**82.50**	**0.652**	**0.906**
Hela	DNA2Vec	77.53	75.48	76.51	0.534	0.853
	OneHot	79.95	74.32	77.13	0.545	0.860
	Positional embedding	**82.25**	**86.51**	**84.38**	**0.690**	**0.919**
Hep-G2	DNA2Vec	80.91	85.14	83.25	0.661	0.908
	OneHot	81.47	84.50	82.98	0.660	0.908
	Positional embedding	**83.00**	**82.58**	82.79	**0.658**	**0.905**
H1-hESC	DNA2Vec	80.19	81.31	**83.25**	0.639	0.896
	OneHot	81.26	82.13	82.72	0.636	0.891

Sen, sensitivity; Spe, specificity; Acc, accuracy; MCC, Mathew’s correlation coefficient; AUC, area under the receiver operating characteristic curve.

The bold section indicates the best performing indicators in each dataset.

As can be seen from **Table 3**, on the five datasets, our positional embedding method obviously has the best performance, always having the highest AUC value and MCC value in the DNA embedding methods. This means that our positional embedding method has significantly better performance than the current popular embedding methods. Beyond that, the OneHot method has better performance in the model on many evaluation values, namely, Sen, Acc, and MCC were 1.47%, 0.276%, and 0.6% higher than has DNA2Vec. However, the best performance is positional embedding, which is higher than the OneHot method in all the evaluation indexes of the five datasets, 1.14%, 2.664%, 1.7%, 0.39%, and 0.16% in Sen, Spe, Acc, MCC, and AUC, respectively.

In our opinion, the reason why the location embedding method can achieve better results is that it combines the advantages of OneHot encoding and DNA2Vec encoding. It has a distributed representation of the content encoded by DNA2Vec, as well as location information in the OneHot encoding. Therefore, with a suitable model, the position embedding method can show better performance.

### Performance Comparison With Other Existing Predictors

**Figure 3:**
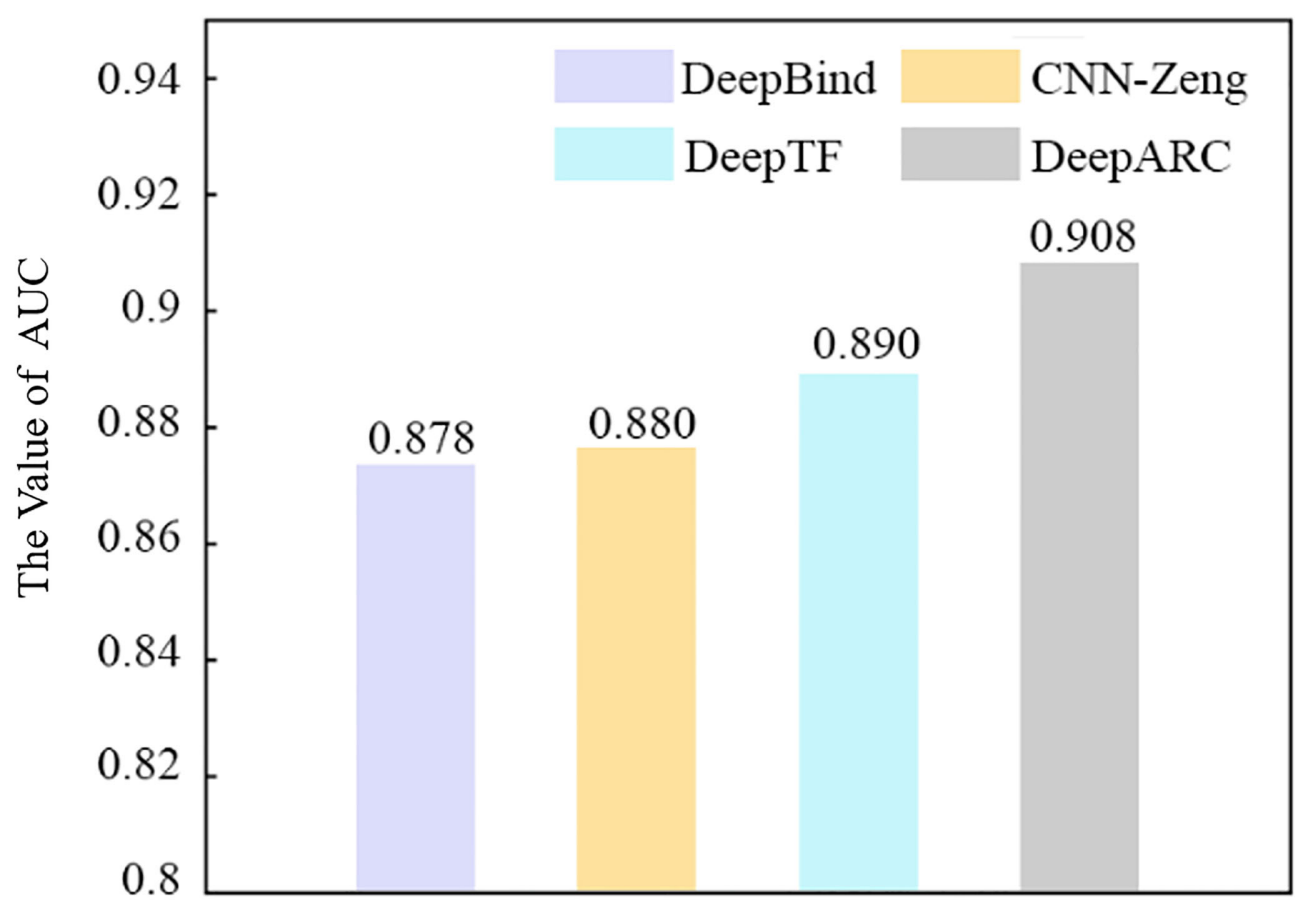
Performance of DeepARC and three existing predictors in ROC-AUC. ROC, receiver operating characteristic; AUC, area under the ROC curve.

In this part, in order to analyze the performance of DeepARC, we compare it with several other prediction methods (DeepTF, DeepBind, and CNN-Zeng) on 50 randomly selected datasets. The comparison results obtained are shown in **Table 4**. As can be seen from the table, among the four methods, DeepARC has the best performance in each evaluation measurement. In addition, compared with DeepTF, which has the best performance among the other competitive two methods, DeepARC has higher Sen, Spe, Acc, and MCC evaluation indexes of 4.58%, 2.83%, 2.12%, and 3.2%, respectively. The average AUC values of each method on five cell lines are shown in **Figure 3**. It can also be seen from **Figure 3** that DeepARC has the highest accuracy and is 1.8% higher than the second-place method DeepTF.

**Table 4:** Performance comparison of DeepARC and three existing predictors.

Model	*Sen* (%)	*Spe* (%)	*Acc* (%)	*MCC*
DeepARC	**82.02**	**84.19**	**83.10**	**0.664**
DeepTF	77.44	81.36	80.98	0.632
CNN-Zeng	72.12	81.96	79.92	0.619
DeepBind	72.64	81.44	79.82	0.609

Sen, sensitivity; Spe, specificity; Acc, accuracy; MCC, Mathew’s correlation coefficient.

The bold part indicates the index with the best performance.

According to the results, DeepARC has better predictive performance on the dataset compared with other methods. By analyzing the model architecture of DeepARC and several other methods, we reasonably believe that the position embedding method and model architecture of DeepARC, especially the use of the attention mechanism, play a promoting role in the experiment, thus improving the prediction performance of the model.

### Attention Mechanism Brings Interpretation

**Figure 4:**
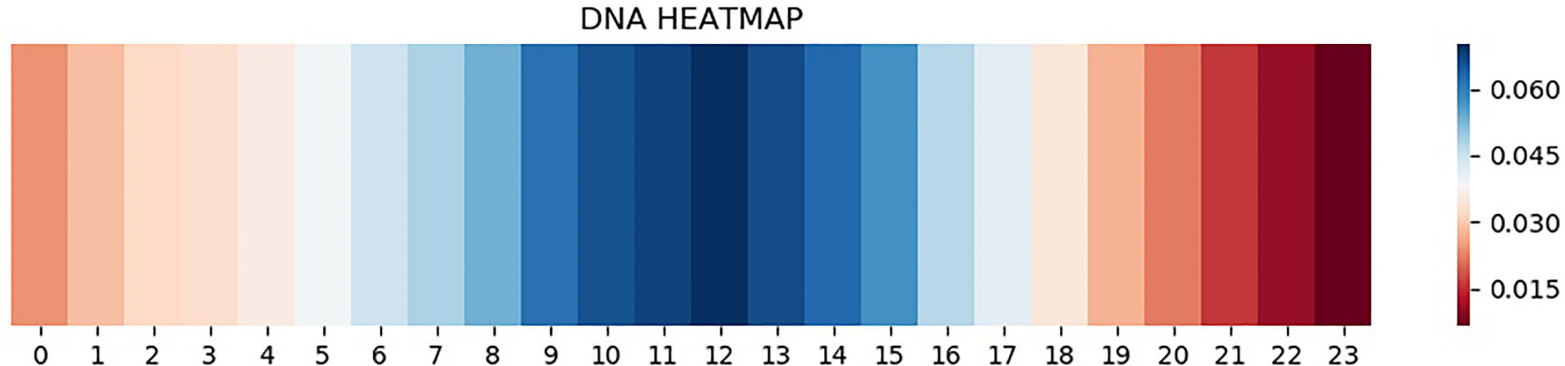
Heatmap of H1-hESC.

**Figure 5:**
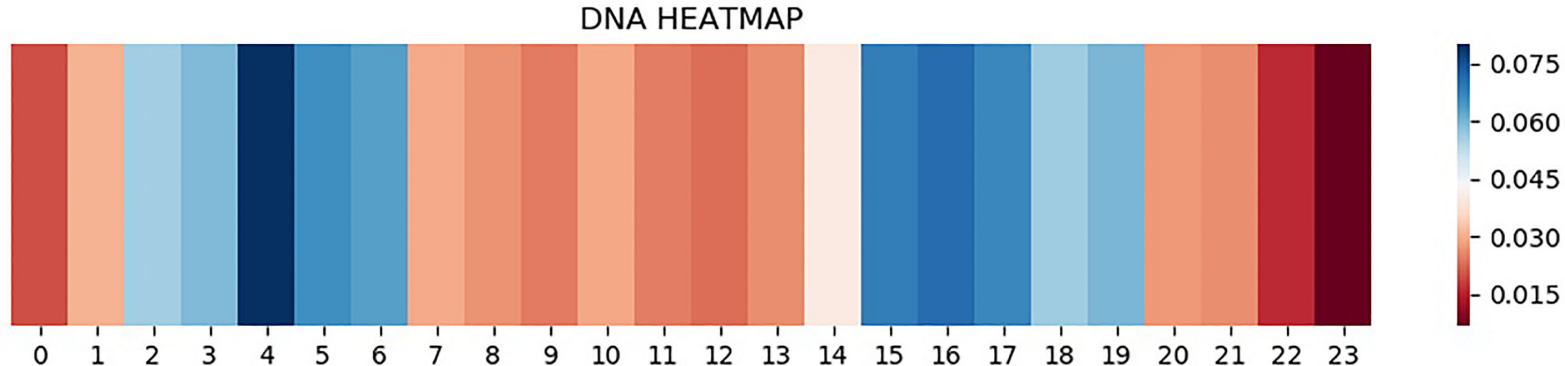
Heatmap of A5493.

CNN is one of the representative deep learning algorithms that are widely used at present, and it has a robust feature learning ability. But because of the high complexity of its architecture, it is often difficult to understand and explain the decisions that these networks make (31–34). Therefore, a layer of attention mechanism was added in DeepARC to enhance the weight of attention on the motif. In addition, we also visually display the average weights of different datasets in the model to strengthen the interpretability of the model (**Figures 4**, **5**).

As can be seen from **Figure 4**, attention is a major concentration in the intermediate region. In other words, the model determines whether there is a TFBS in the input gene information mainly by sensing the peak value of the gene sequence. The theory of peeling existing binding site sequences from the peak is consistent with this. However, there are two attention peaks shown in **Figure 5**, indicating that the model recognizes the presence of two TFBSs in the sequence.

## Conclusions

In this work, we describe a novel attention-based network model named DeepARC to predict TFBSs. Driven by its beneficial strength of combining DNA2Vec and OneHot encoding, DeepARC could embed the gene information into a distributed positional representation and then predict the output using attention-based CNN-BiLSTM network architecture. The comparative work shows that DeepARC is superior to the existing state-of-the-art methods. To demonstrate the interpretability of DeepARC, we visualized the attention weights and found that the attention weight was concentrated in the peak region of the ChIP-seq. Although our method achieves good results, there is still room for improvement. DeepARC only uses DNA sequence information for feature embedding. Evolution information, physical–chemical properties, and embedding from language models can be integrated to improve performance in the future. On the other hand, the attention mechanism can be optimized to mark the accurate TFBS fragments directly.

## Data Availability Statement

The original contributions presented in the study are included in the article/supplementary material. Further inquiries can be directed to the corresponding author.

## Author Contributions

Conceptualization: LC, JC, and LD. Methodology: LC, PL, JC, and LD. Validation: LC, JC, and LD. Writing—original draft preparation, LC, PL, and JC. Writing—review and editing: LD. Supervision: LD. Project administration: LD. Funding acquisition, LD. All authors have read and agreed to the published version of the manuscript.

## Funding

This work was supported by the National Natural Science Foundation of China under grant No. 61972422 and No. 61672541. Publication costs are funded by the National Natural Science Foundation of China under grant No. 61972422. The funding body has not played any role in the design of the study and collection, analysis, and interpretation of data in writing the manuscript.

## Conflict of Interest

The authors declare that the research was conducted in the absence of any commercial or financial relationships that could be construed as a potential conflict of interest.

## Publisher’s Note

All claims expressed in this article are solely those of the authors and do not necessarily represent those of their affiliated organizations, or those of the publisher, the editors and the reviewers. Any product that may be evaluated in this article, or claim that may be made by its manufacturer, is not guaranteed or endorsed by the publisher.
